# Microbial Asymmetric Functionalization of β-Cyclocitral-Derived Tetramethyl-Substituted γ-Lactone

**DOI:** 10.3390/molecules24040666

**Published:** 2019-02-13

**Authors:** Marcelina Mazur, Witold Gładkowski, Aleksandra Pawlak, Bożena Obmińska-Mrukowicz, Gabriela Maciejewska, Czesław Wawrzeńczyk

**Affiliations:** 1 Department of Chemistry, Wrocław University of Environmental and Life Sciences, Norwida 25, 50-375 Wrocław, Poland; glado@poczta.fm (W.G.); czeslaw.wawrzenczyk@upwr.edu.pl (C.W.); 2 Department of Pharmacology and Toxicology, Wrocław University of Environmental and Life Sciences, Norwida 31, 50-375 Wrocław, Poland; aleksandra.pawlak@upwr.edu.pl (A.P.); bozena.obminska-mrukowicz@upwr.edu.pl (B.O.-M.); 3 Central Laboratory of the Instrumental Analysis, Wrocław University of Science and Technology, Wybrzeże Wyspiańskiego 27, 50-370 Wrocław, Poland; gabriela.maciejewska@pwr.edu.pl

**Keywords:** asymmetric hydroxylation, antiproliferative activity, *Fusarium culmorum*, *Armillaria mellea*, *Trametes versicolor*

## Abstract

Searching for the new anticancer compounds we prepared three new β-cyclocitral-derived hydroxyl-γ-lactones by microbial hydroxylation of tetramethyl-substituted bicyclic γ-lactone. The substrate was transformed by the enzymatic system of filamentous fungi. Three out of fifteen strains were selected as effective biocatalysts (*Fusarium culmorum* AM10, *Armillaria mellea* AM296, *Trametes versicolor* AM536). The hydroxylation processes were not only regioselective but also stereoselective. The hydroxylation products of each secondary carbon atom in the cyclohexane ring were obtained by the application of the selected fungal strains. The *Fusarium culmorum* AM10 introduced the hydroxy function at C-3 and C-4, *Armillaria mellea* AM296 incorporated the hydroxy function at C-3 and C-5 and *Trametes versicolor* AM536 transformed the substrate to the mixture of C-3, C-4 and C-5 hydroxylactones. The hydroxylactones obtained were enantiomericaly enriched (ee values in the range 17–99%). The in vitro antiproliferative activities of the functionalization products were also evaluated. Regardless of the hydroxy substituent location all tested lactones exhibited similar, significant activity towards selected cancer cell lines (IC_50_ in the range 22.8–33.9 µg/mL).

## 1. Introduction

There appears to be an increasing occurrence of cancer in animals. In livestock farming, the biggest problems are cattle and poultry cancers. In medical-veterinary practice, cancer is considered to be the main cause of mortality in dogs. Hematopoietic disorders account for approximately 30% of all canine cancers. Dogs may be a useful model for the study of new anticancer drugs and therapeutic strategies for humans because of the similarity of some canine and human cancers in etiology, pathogenesis and response to treatment as well as anatomical and physiological similarities of dogs to humans [1]. In our previous paper, we reported the synthesis of a series of δ-halo-γ-lactones derived from β-cyclocitral [2]. Those compounds did not show any antiproliferative activity towards the selected cancer cell lines in vitro [3]. In our search for new molecules, which are active against canine cancer cell lines, we decided to obtain β-cyclocitral-derived lactones with hydroxy function.

Natural hydroxylactones are important secondary plant metabolites that exhibit a wide range of biological activities such as antioxidant, anti-inflammatory, antimicrobial or antiviral [4,5,6,7]. Among them, cytotoxic activity is at the forefront [8,9,10]. The synthetic hydroxylactones are known to be antibacterial and antifungal agents [11,12,13]. They also exhibit antifeedant properties [14] or cytotoxic activity [15,16]. Numerous examples confirm that the introduction of hydroxyl function into the lactone structure increases biological activity [14,17,18,19]. The most commonly used methods to obtain the chiral alcohols are asymmetric reduction of ketones or the lipase-catalyzed kinetic resolution of racemic alcohols. Nevertheless, these methods require hydroxyl or carbonyl groups in a defined place in a compound structure. The microbial hydroxylation is known to be regio and enantioselective and the use of biocatalysts makes it possible to introduce the hydroxyl function to unactivated carbon atoms. The hydroxylation potential of filamentous fungi has been applied by the pharmaceutical industry to develop products such as hydroxylated flavonoids [20,21] and steroid compounds [22,23,24]. There are also documented examples of bicyclic γ-lactone hydroxylation catalysed by filamentous fungi [3,25]. In our research we selected three fungal strains (*Fusarium culmorum* AM10, *Armillaria mellea* AM296, *Trametes versicolor* AM536) with the ability for selective hydroxylation of tetramethyl-γ-lactone (**1**). All selected microorganisms are already known for their hydroxylation potential towards steroid compounds [26], terpenes [27,28,29,30] or pesticides [31].

## 2. Results and Discussion

The strains selection was based on screening tests. The metabolism of lactone **1** in the cultures of fifteen filamentous fungi strains was continually monitored during fourteen days. Three of the tested microorganisms (*Fusarium culmorum* AM10, *Armillaria mellea* AM296, *Trametes versicolor* AM536) efficiently transformed the substrate. The results of the screening experiments, as a composition of the products mixture, are presented in Table 1. The selected biocatalysts showed differences in the transformation rate as well as the products’ composition. In all experiments, the formation of hydroxylated derivatives was observed to vary in the substituent location. It is worth noting, that we were able to obtain the derivative with the hydroxy group located at each unactivated position of the cyclohexane ring (C-3, C-4, C-5). In the cultures of both the *Fusarium culmorum* AM10, and the *Armillaria mellea* AM296, two hydroxylated metabolites were formed; in the culture of *Trametes versicolor* AM536 three hydroxylated metabolites were formed. *Armillaria mellea* AM296 transformed the substrate **1** with the highest rate, while the slowest reaction was observed for *Trametes versicolor* AM536.

**Scheme 1:**
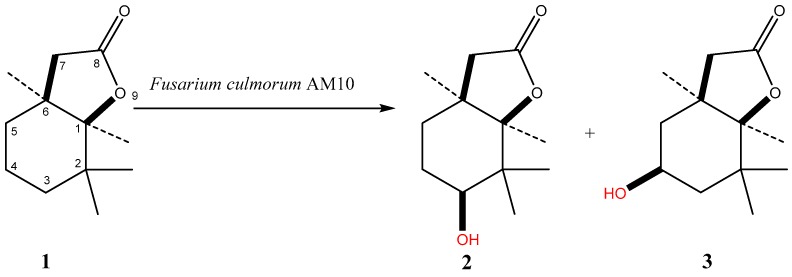
Biohydroxylation of lactone **1**. The structures present relative stereochemistry.

**Figure 1:**
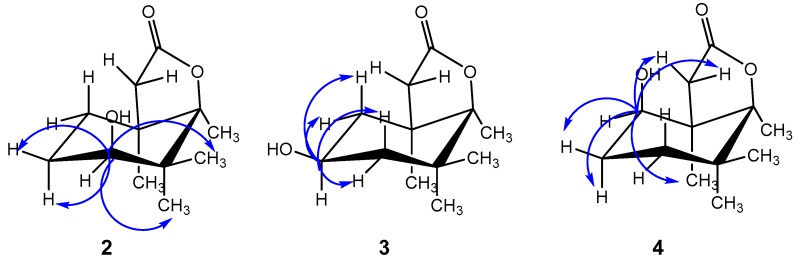
Determination of the hydroxyl function location using relevant H-C correlations from the HMBC spectra.

Biotransformationn of lactone **1** in *Fusarium culmorum* AM10 culture after 10 days led to C-3 (**2**) and C-4 (**3**) hydroxylation products (Scheme 1). The structures of the products were established on the basis of spectroscopic data (Supplementary materials Figures S1–S8). The IR spectrum of lactone **2** showed absorption bands at 3512 cm^−1^ and 1751 cm^−1^, which proved the incorporation of the hydroxy group as well as retention of the γ-lactone ring. The NMR analysis, especially the correlation spectroscopy (Figure 1) (HMBC and HSQC), was crucial to prove the location and orientation of the hydroxy function. On the HMBC spectrum the correlation between the protons of gem-dimethyl substituent and the signal of C-3 at 73.26 ppm could be observed. This correlation confirmed the incorporation of the hydroxy group at C-3. On the ^1^H-NMR spectrum the triplet from H-3 was present at 3.67 ppm. The coupling constant value (*J* = 6.8 Hz) found for this signal, indicated the equatorial orientation for this proton and in consequence the axial orientation of the hydroxy group in cis relation to γ-lactone ring. The second transformation product was the 4-hydroxy derivative (**3**) with the OH group located cis to the γ-lactone moiety. Evident similarities to 3-hydroxy-γ-lactone, like absorption bands at 1764 cm^−1^ and 3415 cm^−1^ on the IR spectrum, were listed. However, the NMR analysis revealed substantial differences. On the COSY spectrum the signal shifted to the lower field (3.87 ppm) was correlated with CH_2_-3 and CH_2_-4 protons. Simultaneously the absence of correlation between this signal and the carbon atoms of the CH_3_ groups at C-2 (HMBC spectrum), can be explained only when the hydroxy substituent is located at C-4. Considering the shape of the H-4 multiplet and analyzing the signals from the CH_2_-5 protons it was possible to assign the orientation of the hydroxy function. The high coupling constant (*J* = 13.0 Hz), present in the triplet from one of the CH_2_-5 protons (1.37 ppm) and the shape of the multiplet at 3.87 ppm, suggests the axial orientation of the H-4 proton and as a consequence, the equatorial position of the hydroxy function. The chiral GC analyses were performed to determine the enantiomeric excess of all the hydroxylated derivatives. Both products were obtained as enantiomerically enriched (+)-isomeres, the lactone **2** was obtained with ee = 92% and the 4-hydroxylactone **3** with lower enantiomeric excess (31%).

**Scheme 2:**
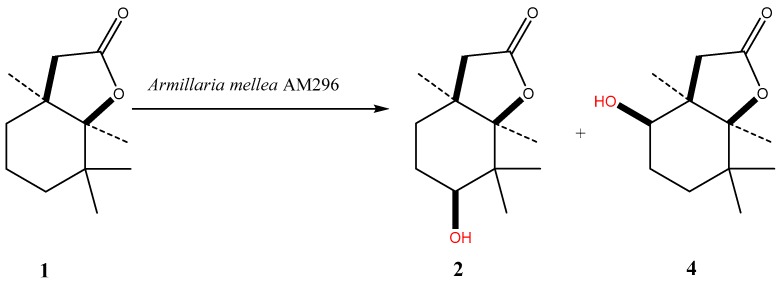
Biotransformation of lactone **1** in *A. mellea* AM296 culture. The structures present relative stereochemistry.

The products obtained after seven days of transformation in the *Armillaria mellea* AM296 culture were C-3 and C-5 hydroxy-γ-lactones (**2** and **4**) (Scheme 2). The IR analysis of product **4** revealed the presence of characteristic C=O absorption bands at 1758 cm^−1^ and O-H bond stretching vibration at 3518 cm^−1^. As for the previous products, the position of the hydroxy group was established on the basis of NMR analysis (Supplementary materials Figures S9–S12). On the HMBC spectrum the evident correlation between the signal of C-5 at 73.77 ppm and signals of CH_2_-7 protons and methyl group CH_3_-6 indicated the C-5 hydroxylation. Similarly to the 3-hydroxy derivative **2**, the H-5 signal shift to the lower field by the deshielding effect of oxygen was confusingly similar to the triplet. Its shape points to the equatorial position of this proton and consequent axial orientation of the hydroxy group, cis in relation to the γ-lactone ring. The highest conversion of the substrate was observed in the *A. mellea* culture and the products were obtained with high or moderate enantiomeric excess (99% of the (+)-isomer of the 3-hydroxylactone **2** and ee = 65% for the (–)-isomer of 5-hydroxylactone **4**).

Interestingly, for both the products with axial orientation of the hydroxy group it is possible to form a hydrogen bond between the alkoxy oxygen in the γ-lactone ring and the hydrogen atom of the hydroxy group. This weak binding can have a positive effect on the increased conformational stability for the 3- and 5-hydroxy derivatives and can prevent inversion of the ring to the one in which the hydroxy function occupies equatorial position.

**Scheme 3:**
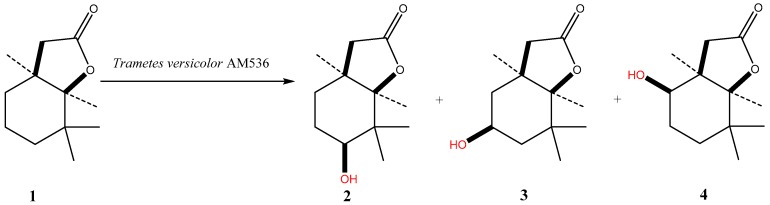
Products mixtures obtained in *T. versicolor* AM536 culture. The structures present relative stereochemistry.

The twelve days biotransformation of lactone **1** in *Trametes versicolor* AM536 culture was the least selective and resulted in three hydroxylation products (**2**, **3**, **4**) described below (Scheme 3). All three hydroxylactones were formed at the same time but the 3-hydroxylactone was the main product in the mixture (15% isolated yield). The high enantiomeric excess was determined for the 3-hydroxylactone **2** (82%, (+)-isomer), and the lowest ee = 17% was obtained for the (–)-isomer of 5-hydroxylactone **4**. The product with the lowest percentage in the products mixture was 4-hydroxylactone **3** but it exhibited the highest enantiomeric excess (ee = 96%) of the (–)-isomer.

All obtained hydroxyderivatives (**2**–**4**) were subjected to in vitro antiproliferative tests. In the MTT test, two cell lines (GL-1 and CLB 70) representing different types of leukemia in dogs were used. Leukemia is a significant problem in the dog population and there remains a lack of effective therapies. In addition, previous studies have shown that canine hematopoietic cancer cells are sensitive to the effects of various compounds with γ-lactone rings [32,33] and that there is a relationship between a configuration of chiral centers of the selected lactones and their anticancer activity [34,35].

The results are presented in Table 2 as IC_50_ values (IC_50_—anticancer drug concentration inhibiting cell proliferation by 50%). Regardless of the hydroxy group position, all lactones exhibited very similar antiproliferative potential. The tested compounds did not exhibit statistical differences in activity related to the hydroxyl function placement. However, the GL-1 cell line was slightly more sensitive towards the tested compounds. Considering the IC_50_ values towards both cancer cell lines tested the lactones have significant antiproliferative effect, comparable with other γ-lactones [32,33].

The worldwide study on anticancer drugs revealed complex interactions. The anticancer activity can be associated with the induction of apoptosis and inhibition of STAT3 and NF-κB transcription factors activation via oxidative stress [36,37]. For numerous sesquiterpene lactones the cytotoxic activity is related to the presence of α-methylene group in the γ-lactone ring or α,β-unsaturated ketone which can function as a Michael acceptor and interact through nucleophilic attack with the SH group of proteins, GSH and nitrogen bases, mainly guanine [36,38]. In our previous work we presented the results of antitumor activity exhibited by β-aryl-δ-iodo-γ-lactones, which induced apoptosis via a mitochondrial-mediated, caspase-dependent pathway [35]. The mechanism of cytotoxic activity of hydroxylactones **2**–**4** can be similar but further, in-depth studies are required in this area.

## 3. Material and Methods

### 3.1. Analysis

The progress of transformations and the purity of isolated products were monitored by TLC (silica gel on aluminium plates, DC-Alufolien Kieselgel 60 F_254_, Merck, Darmstadt, Germany) and gas chromatography. GC analysis was performed on the Agilent Technologies 6890N instrument (Santa Clara, CA, USA) using Agilent DB-5HT capillary column ((50%-phenyl)-methylpolysiloxane 30 m × 0.25 mm × 0.10 μm) and hydrogen as the carrier gas. The temperature programme was as follows: injector 210 °C, detector (FID) 280 °C, column temperature: 80–200 °C (rate 25 °C min^−1^), 200–300 °C (rate 30 °C min^−1^), 300 °C (1 min). The enantiomeric excesses of biotransformation products were calculated on the basis of chiral GC analysis using CP Chirasil-Dex CB column (25 m × 0.25 mm × 0.25 μm) at the following conditions: injector 210 °C, detector (FID) 280 °C, column temperature: 80 °C (hold 0 min), 80–160 °C (rate 0.3 °C min^−1^), 160–200 (rate 20 °C min^−1^), 200 °C (hold 1 min).

The biotransformation products were purified by column chromatography on silica gel (Kieselgel 60, 230–400 mesh, Merck).

The NMR spectra (^1^H, ^13^C-NMR and correlation spectra: ^1^H-^1^H COSY, ^1^H-^13^C HMQC, ^1^H-^13^C HMBC) were recorded in a CDCl_3_ solution on a Brüker Avance DRX 300 MHz spectrometer. Residual solvent signals (δH = 7.26, δC = 77.16) were used as references for chemical shifts.

IR spectra were determined using Mattson IR 300 Thermo Nicolet Spectrophotometer (Waltham, MA, USA). The melting points (uncorrected) were determined on a Boetius apparatus. Optical rotations were measured on a Jasco P-2000 Digital Polarimeter (version with iRM controller, Easton, MD, USA).

High-resolution mass spectra (HRMS) were recorded using electrospray ionization (ESI) technique on a Waters ESI-QTOF Premier XE Spectrometer (Waters Corp., Millford, MA, USA).

### 3.2. Substrates for Biotransformation

Racemic bicyclic tetramethyl-substituted γ-lactone (**1**) was obtained from β-cyclocitral in five step chemical synthesis, according to the procedure described earlier [2].

### 3.3. Microbial Transformations

Fifteen strains of filamentous fungi used in this work came from the collection of the Institute of Biology and Botany, Wrocław Medical University (AM) and from the collection of Department of Phytopathology, University of Agriculture in Kraków (AR) (*Nigrospora oryzae* AM8, *Fusarium culmorum* AM10, *Fusarium avenaceum* AM12, *Cenangium ferruginosum* AR56, *Penicillium camembertii* AM83, *Absidia coerulea* AM93, *Syncephalastrum racemosum* AM105, *Mortierella vinaceae* AM149, *Mortierella isabellina* AM212, *Absidia glauca* AM254, *Beauveria bassiana* AM278, *Armillaria mellea* AM296, *Absidia cylindrospora* AM336, *Laetiporus sulphurens* AM524, *Trametes versicolor* AM536). Cultivation of microorganisms was carried out on Sabouraud agar slants of the following composition: glucose (40.0 g), peptone K (5.0 g), and aminobac (5.0 g) in distilled water (1 L). The microorganisms were cultivated at 28 °C and stored in a refrigerator at 4 °C.

### 3.4. Screening Procedure

The strains were cultivated at 25 °C in 300 mL Erlenmeyer flasks containing 50 mL of medium (3% glucose, 0.5% peptone K, 0.5% aminobac in distilled water). After 3 days of growth 10 mg of lactone (**1**) dissolved in 1 mL of acetone were added to the shaken cultures (170 rpm). The incubation was carried out for 14 days. After 2, 4, 7, 10, 12 and 14 days of incubation, the products were extracted with methylene chloride and analyzed by TLC and GC.

### 3.5. Biotransformation

Three strains of fungi *(Fusarium culmorum* AM10, *Armillaria mellea* AM296, *Trametes versicolor* AM536) have been selected as effective biocatalysts for transformation of lactone (**1**). For isolation and identification of products, the biotransformations were performed in multiplied scales. Selected filamentous fungi strains were cultivated in 10 Erlenmeyer flasks. After 3 days of growth 10 mg of substrate (**1**) dissolved in 1 mL of acetone were added to each flask (total amount 100 mg, reaction conditions were the same as described in screening procedure). After the optimal time for each biotransformation (*Fusarium culmorum* AM10—10 days, *Armillaria mellea* AM296—7 days, *Trametes versicolor* AM536—12 days) the products were extracted two times with methylene chloride (50 mL for each flask). The organic layers were pooled, dried over anhydrous MgSO_4_ and the solvent was evaporated in vacuo. The transformation products were separated and purified by column chromatography (hexane:acetone:isopropanol starting from 15:1:1 followed by 10:1:1). Biotransformation products from *Fusarium culmorum* AM10 culture were 3-hydroxylactone (**2**) (19 mg, 18% isolated yield) and 4-hydroxylactone (**3**) (20 mg, 19% yield). *Armillaria mellea* AM296 transformed the substrate (**1**) to 3-hydroxylactone (**2**) (36 mg, 33% isolated yield) and 5-hydroxylactone (**4**) (17 mg 16% isolated yield). The biotransformation by *Trametes versicolor* AM536 culture afforded three products: 3-hydroxylactone (**2**) (16 mg, 15% isolated yield), 4-hydroxylactone (**3**) (4 mg, 4% isolated yield) and 5-hydroxylactone (**4**) (5 mg, 5% isolated yield). The physical and spectral data of the biotransformation products are given below.

*(+)-rel(1R,3S,6S)-3-Hydroxy–1,2,2,4-tetramethyl-9-oxabicyclo[4.3.0]nonan-8-one* (**2**), White crystals, m.p. = 131–138 °C; $\alpha_{D}^{25}$ = +10.4 (c = 1.8, CHCl_3_, ee = 92%); ^1^H-NMR (300 MHz, CDCl_3_) δ: 0.96 and 1.17 (two s, 6H, (C*H*_3_)_2_C<), 1.22 (s, 3H, C*H*_3_-6), 1.34 (s, 3H, C*H*_3_-1), 1.57–1.67 (m, 5H, C*H*_2_-4, C*H*_2_-5 and -O*H*), 2.12 and 2.61 (two d, *J* = 17.0 Hz, 2H, C*H*_2_-7), 3.67 (t, *J* = 6.8 Hz, 1H, *H*-3); ^13^C-NMR (75 MHz, CDCl_3_) δ: 16.87 and 21.83 ((*C*H_3_)_2_C<), 17.88 (*C*H_3_-1), 21.35 (*C*H_3_-6), 26.27 (*C*-4), 34.98 (*C*-5), 41.46 (*C*-6), 41.87 (*C*-2), 46.28 (*C*-7), 73.26 (*C*-3), 93.51 (*C*-1), 175.47 (*C*-8); IR (KBr, cm^−1^): 3512 (s), 1751 (s), 1271 (m), 1006 (m); HRMS (ESI-TOF) *m*/*z* [M + Na]^+^ calcd for C_12_H_20_O_3_ 235.1310; found 235.1311; GC t_r_ = 5.343; CGC t_r_ = 209.29, 213.91.

*(+)-rel-(1R,3R,6R)-4-Hydroxy-1,2,2,4-tetramethyl-9-oxabicyclo[4.3.0]nonan-8-one* (**3**), White crystals, m.p. = 102–106 °C; $\alpha_{D}^{25}$ = +2.1 (c = 1.0, CHCl_3_, ee = 31%); ^1^H-NMR (300 MHz, CDCl_3_) δ: 1.05 and 1.10 (two s, 6H, (C*H*_3_)_2_C<), 1.24 (s, 3H, C*H*_3_-6), 1.29 (s, 3H, C*H*_3_-1), 1.37 (t, *J* = 13.0 Hz, 1H, one of C*H*_2_-5), 1.57 (m, 2H, C*H*_2_-3), 1.86 (dd, *J* = 13.0 and 3.2 Hz, 1H, one of C*H*_2_-5),1.79 (s, 1H, -O*H*) 2.19 and 2.63 (two d, *J* = 17.0 Hz, 2H, C*H*_2_-7), 3.87 (m, 1H, *H*-4); ^13^C-NMR (75 MHz, CDCl_3_) δ: 17.10 (*C*H_3_-1), 22.18 (*C*H_3_-6), 25.77 and 26.99 ((*C*H_3_)_2_C<), 38.51 (*C*-2), 43.59 (*C*-6), 45.75 (*C*-5), 45.88 (*C*-3), 46.63 (*C*-7), 63.14 (*C*-4), 90.70 (*C*-1), 175.31 (*C*-8); IR (KBr, cm^−1^): 3415 (s), 1764 (s), 1267 (m), 1039 (m); HRMS (ESI-TOF) *m*/*z* [M + Na]^+^ calcd for C_12_H_20_O_3_ 235.1310; found 235.1310; GC t_r_ = 5.361; CGC t_r_ = 215.02, 217.54.

*(−)-rel-(1R,3S,6R)-5-Hydroxy-1,2,2,4-tetramethyl-9-oxabicyclo[4.3.0]nonan-8-one* (**4**), White crystals, m.p. = 93–97 °C; $\alpha_{D}^{25}$ = −11.3 (c = 0.85, CHCl_3_, ee = 65%); ^1^H-NMR (300 MHz, CDCl_3_) δ: 1.02 and 1.06 (two s, 6H, (C*H*_3_)_2_C<), 1.15 (s, 3H, C*H*_3_-6), 1.32 (s, 3H, C*H*_3_-1), 1.35 (m, 1H, one of CH_2_-4), 1.55–1.75 (m, 4H, C*H*_2_-3 and one of C*H*_2_-4, -O*H*), 2.50 and 2.68 (two d, *J* =17.4 Hz, 2H, C*H*_2_-7), 3.50 (m, 1H, *H*-5); ^13^C-NMR (75 MHz, CDCl_3_) δ: 14.01 (*C*H_3_-6), 18.29 (*C*H_3_-1), 25.36 and 26.80 ((*C*H_3_)_2_C<), 27.13 (*C*-4), 35.59 (*C*-3), 36.40 (*C*-2), 43.11 (*C*-7), 47.44 (*C*-6), 73.77 (*C*-5), 93.48 (*C*-1), 176.11 (*C*-8); IR (KBr, cm^−1^): 3518 (s), 1758 (s), 1269 (m), 1034 (m); HRMS (ESI-TOF) *m*/*z* [M + Na]^+^ calcd for C_12_H_20_O_3_ 235.1310; found 235.1311; GC t_r_ = 5.417; CGC t_r_ = 217.56, 218.61.

### 3.6. Antiproliferative Activity

The GL-1 (canine B-cell leukemia) was obtained from Yasuhito Fujino and Hajime Tsujimoto from the Department of Veterinary Internal Medicine University of Tokyo [39], the CLB 70 (canine B-cell chronic leukemia) was established by Pawlak et al. [40]. The GL-1 cells were maintained in RPMI 1640 culture medium supplemented with 2 mM l-glutamine, 100 U/mL penicillin and 100 µg/mL streptomycin and 10% fetal bovine serum (FBS) (Sigma-Aldrich, Stainheim, Germany). The CLB70 cell line was maintained in Advanced RPMI (Gibco) culture medium supplemented with 100 U/mL penicillin and 100 µg/mL streptomycin and 10% heat-inactivated FBS. Cells were cultured in the incubator with 5% CO_2_ and 95% humidified atmosphere at 37 °C. The antiproliferative assay was performed according to the procedure described earlier [33]. The tested substances were prepared within a concentration range of 6.25–50 µg/mL in the culture medium (DMSO concentration was less than 1% in each dilution). The optical density of formed formazan in untreated control cells was taken as 100%. Viability of test samples was determined as: % Viability = (average OD for test group/average OD for control group) × 100. The results were obtained from more than 3 independent experiments (four wells each) and expressed as mean IC_50_ value ± SD (Table 2). Statistical differences were analyzed using one-way ANOVA. The results were considered significant when *p* < 0.05.

## 4. Conclusions

Three fungal strains were selected to transform tetramethyl-γ-lactone **1**. Application of microorganisms made it possible to obtain the hydroxyderivatives in one step, which would be difficult to achieve by traditional chemical synthesis. In all biotransformation processes the unactivated methylene carbon atoms (C-3, C-4, C-5) were hydroxylated to give products with OH function in cis orientation to the γ-lactone moiety. All isolated hydroxylactones (**2**, **3**, **4**) to the best of our knowledge were not described in earlier literature. What is also important is that the hydroxylactones **2** and **3** were obtained almost as the single enantiomers, ee values 99% and 96% respectively. The results of antiproliferative assay indicate the significant activity of obtained products towards tested canine cancer cell lines. The hydroxyl group position did not have any influence on antiproliferative activity. All hydroxylactones were slightly more active towards the GL-1 cell line in comparison to CLB70. The antiproliferative activity of hydroxylactones towards leukemic lymphocytes seems promising, therefore further studies to determine the possible mechanism of action for this group of compounds are required.

## Figures and Tables

**Table 1:** The composition (in % according to GC) of the products’ mixtures of screening biotransformations of lactone **1**.

Strain	Time of Incubation (days)	Lactone 1	Biotransformation Products		
			2	3	4
*Fusarium culmorum* AM10	2	35	28	37	-
	4	19	32	49	-
	7	7	41	52	-
	10	1	45	53	-
	12	0	46	53	-
*Armillaria mellea* AM296	2	41	53	-	6
	4	13	70	-	17
	7	0	75	-	25
*Trametes versicolor* AM536	2	55	31	3	11
	4	20	59	6	15
	7	9	68	6	17
	10	3	71	7	19
	12	2	71	8	19
	14	2	72	8	18

**Table 2:** The antiproliferative activity of hydroxylactones **2**, **3**, **4** and control – Etoposide against the selected cancer cell lines expressed as IC_50_.

Cell Line	Compound (IC_50_ µg/mL)			
	2	3	4	Etoposide
CLB 70	33.21 ± 2.14 a^1^	32.43 ± 1.72 a	33.97 ± 3.66 a	14.31 ± 2.83
GL-1	28.67 ± 1.91 a	26.76 ± 5.65 a	22.75 ± 1.27 a	4.4 ± 1.14

a ^1^The values in the same row, followed by the same letter, are not statistically different (*p* < 0.05).

## Supplementary Materials

The following are available online, Figure S1: ^1^H-NMR of lactone **2**, Figure S2: ^13^C-NMR of lactone **2**, Figure S3: HMQC spectrum of lactone **2**, Figure S4: HMBC spectrum of lactone **2**, Figure S5: ^1^H-NMR of lactone **3**, Figure S6: ^13^C-NMR of lactone **3**, Figure S7: HMQC spectrum of lactone **3**, Figure S8: HMBC spectrum of lactone **3**, Figure S9: ^1^H-NMR of lactone **4**, Figure S10: ^13^C-NMR of lactone **4**, Figure S11: HMQC spectrum of lactone **4**, Figure S12: HMBC spectrum of lactone **4**.
