# Supplementary material for: Microbial Asymmetric Functionalization of β-Cyclocitral-Derived Tetramethyl-Substituted γ-Lactone
Source: Molecules. 2019 Feb 13;24(4):666. doi: 10.3390/molecules24040666 (PMC6412764; doi:10.3390/molecules24040666)
Supplement: Supplementary file 1 [file molecules-24-00666-s001.pdf]

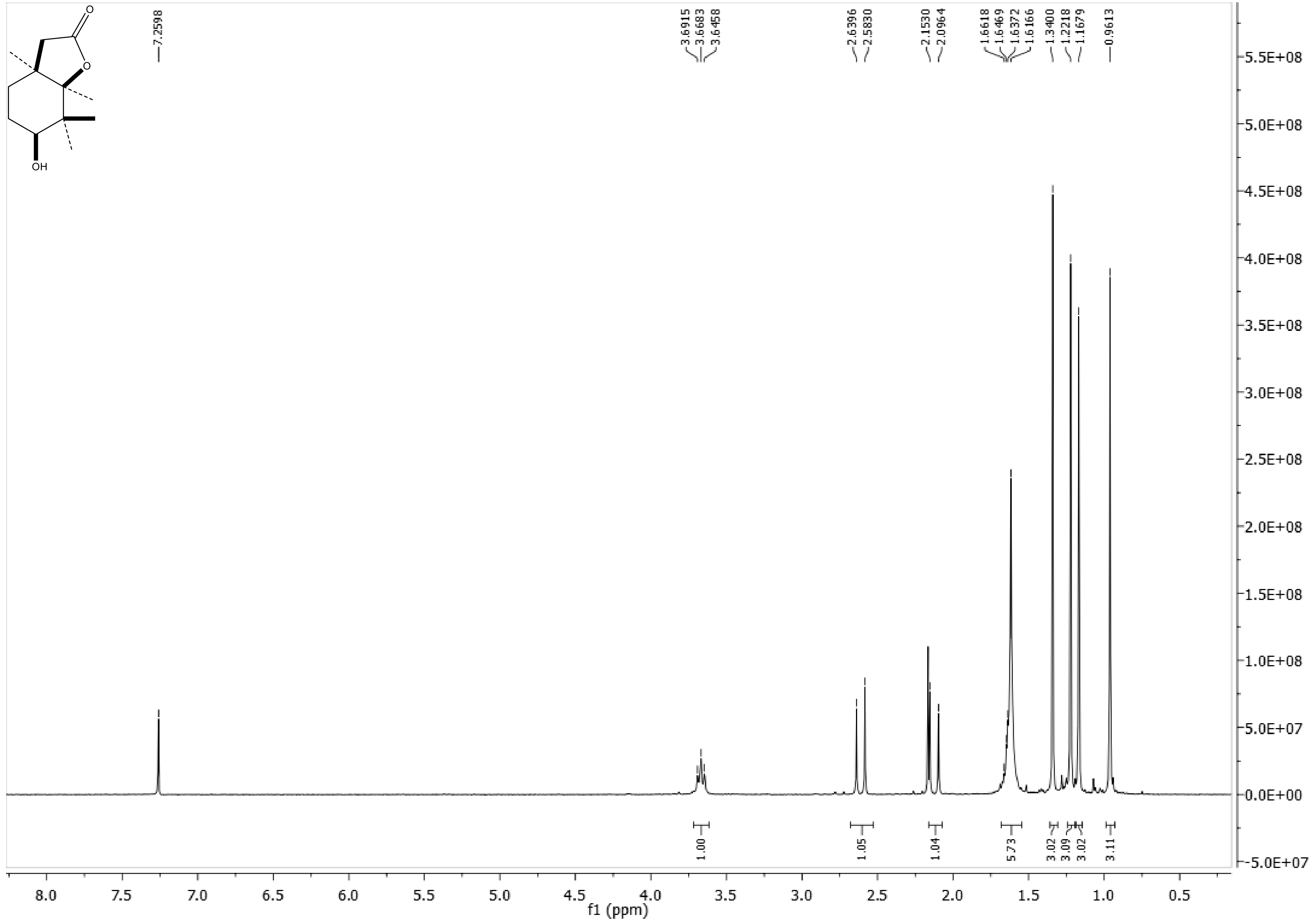

Figure S1. ^{1}H NMR lactone 2

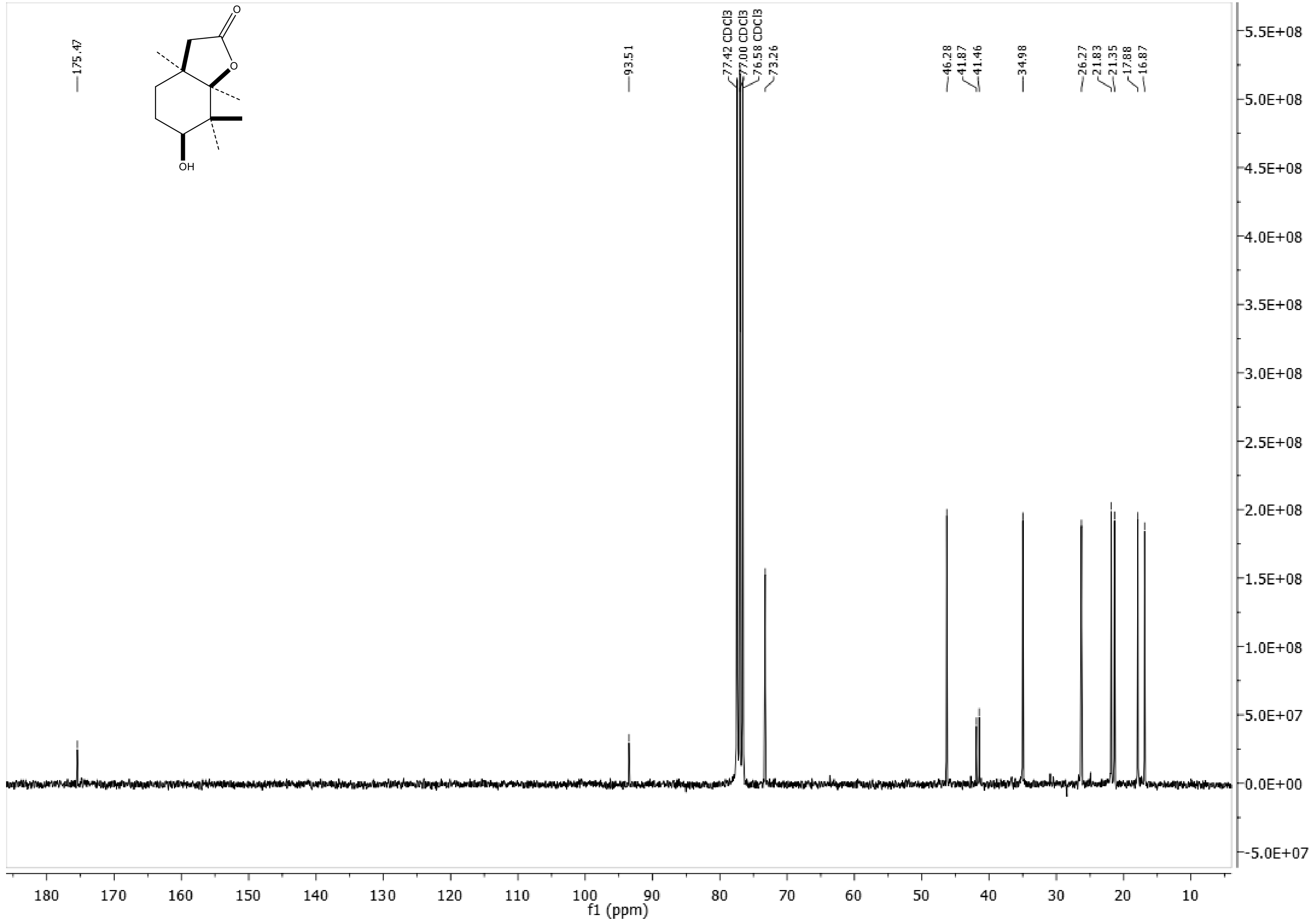

Figure S2. ^{13}C NMR lactone 2

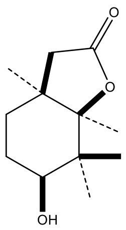

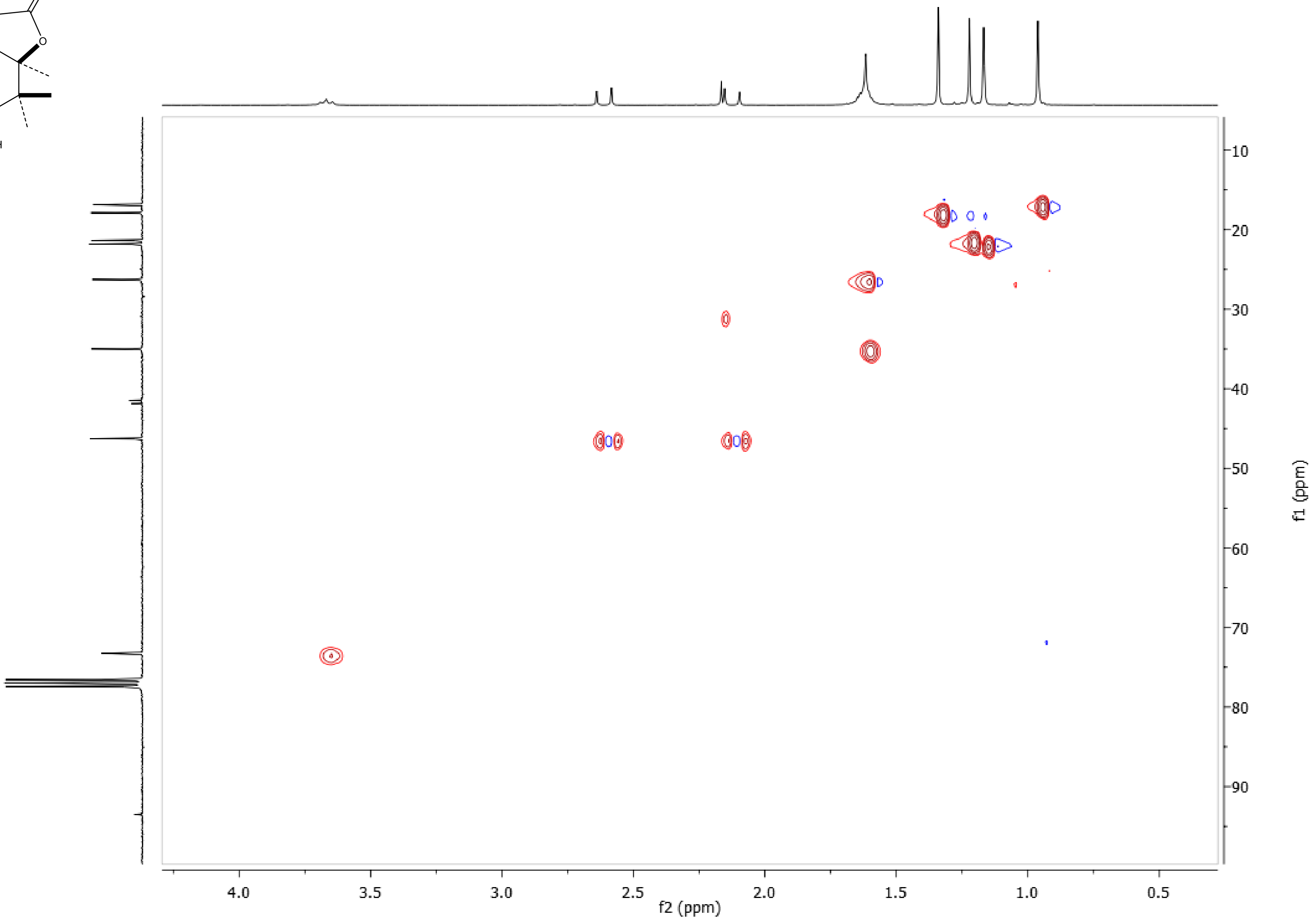

Figure S3. HMQC lactone 2

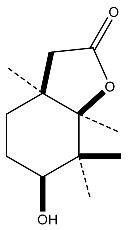

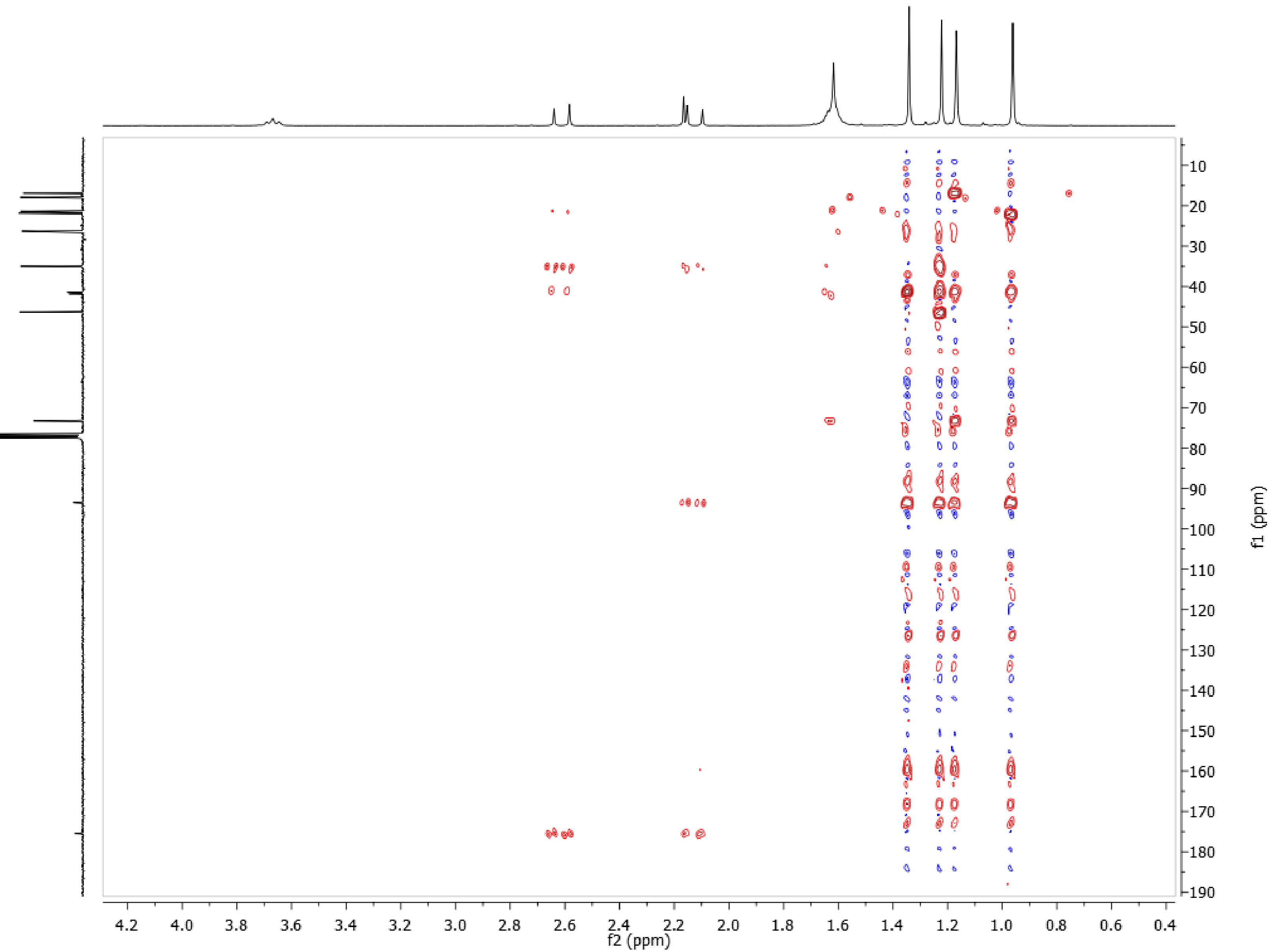

Figure S4. HMBC lactone 2

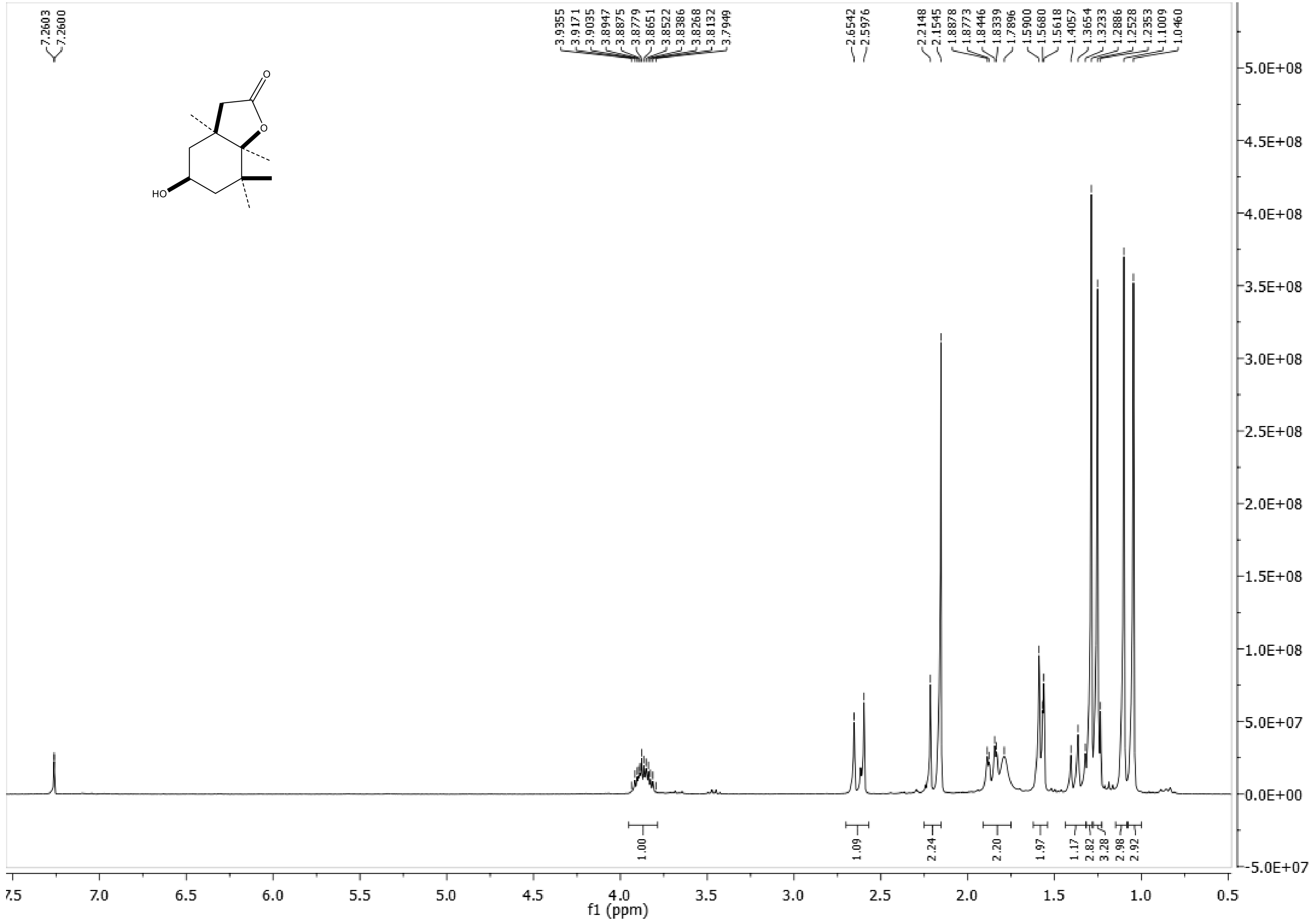

Figure S5. ^{1}H NMR lactone 3

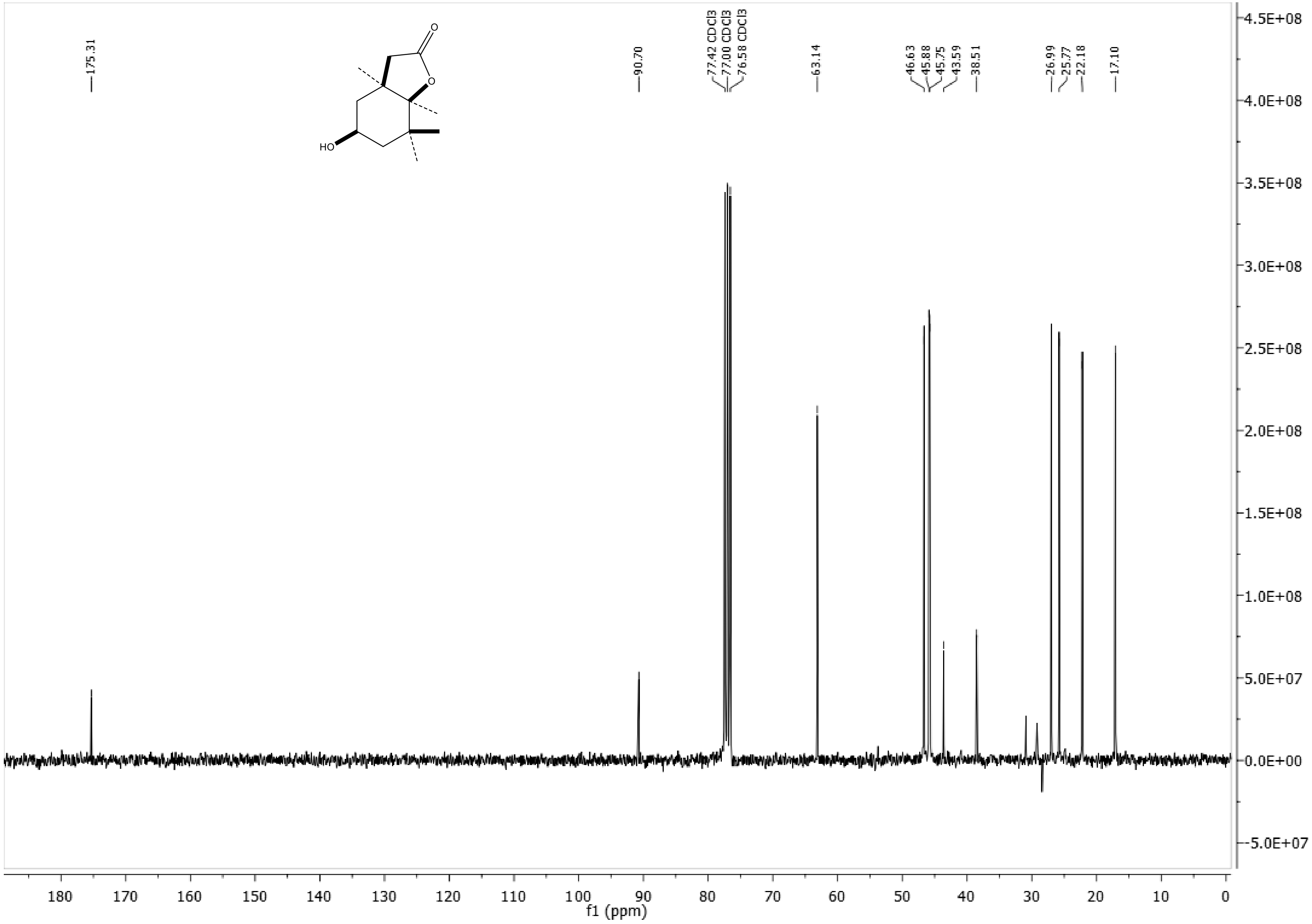

Figure S6. ^{13}C NMR lactone 3

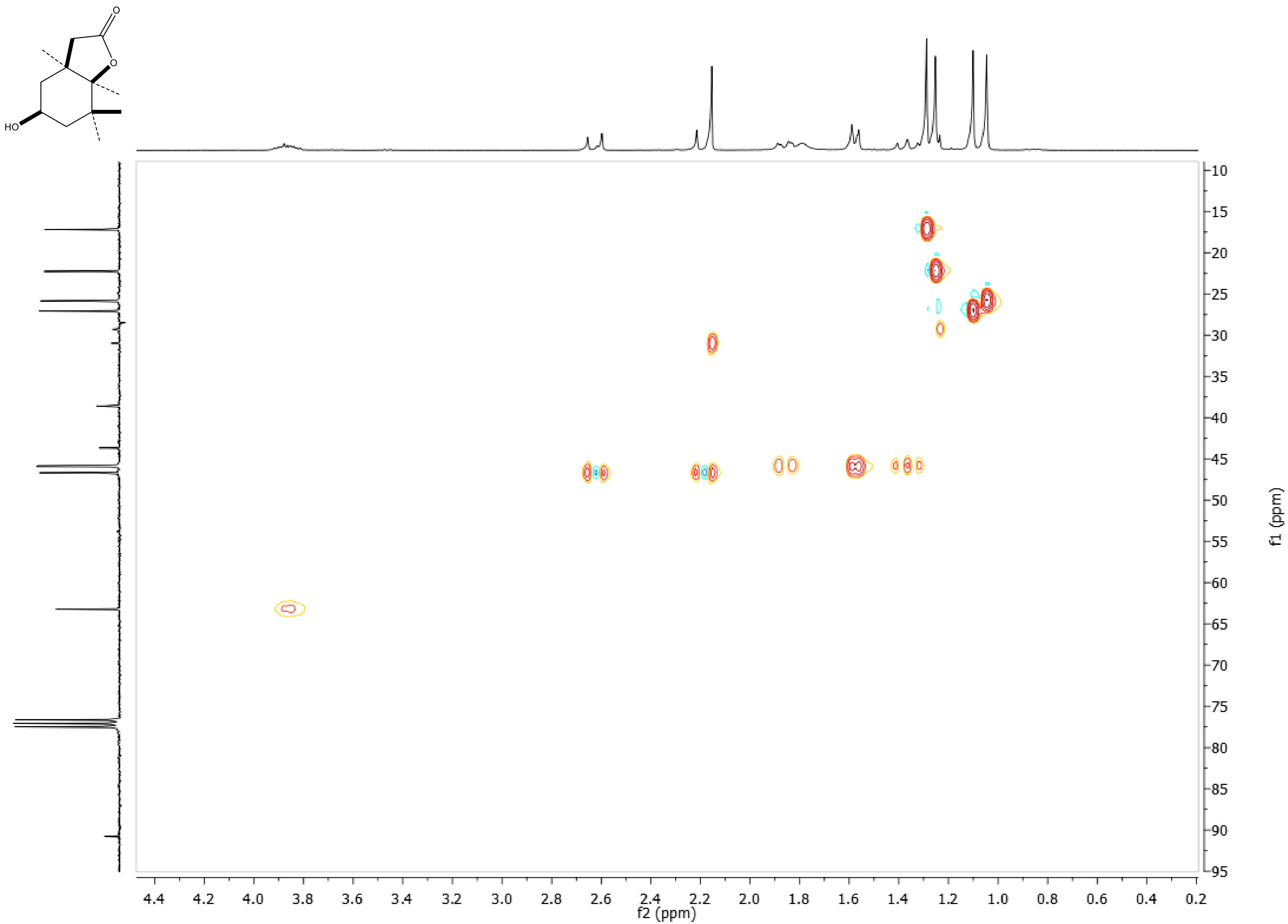

Figure S7. HMQC lactone 3

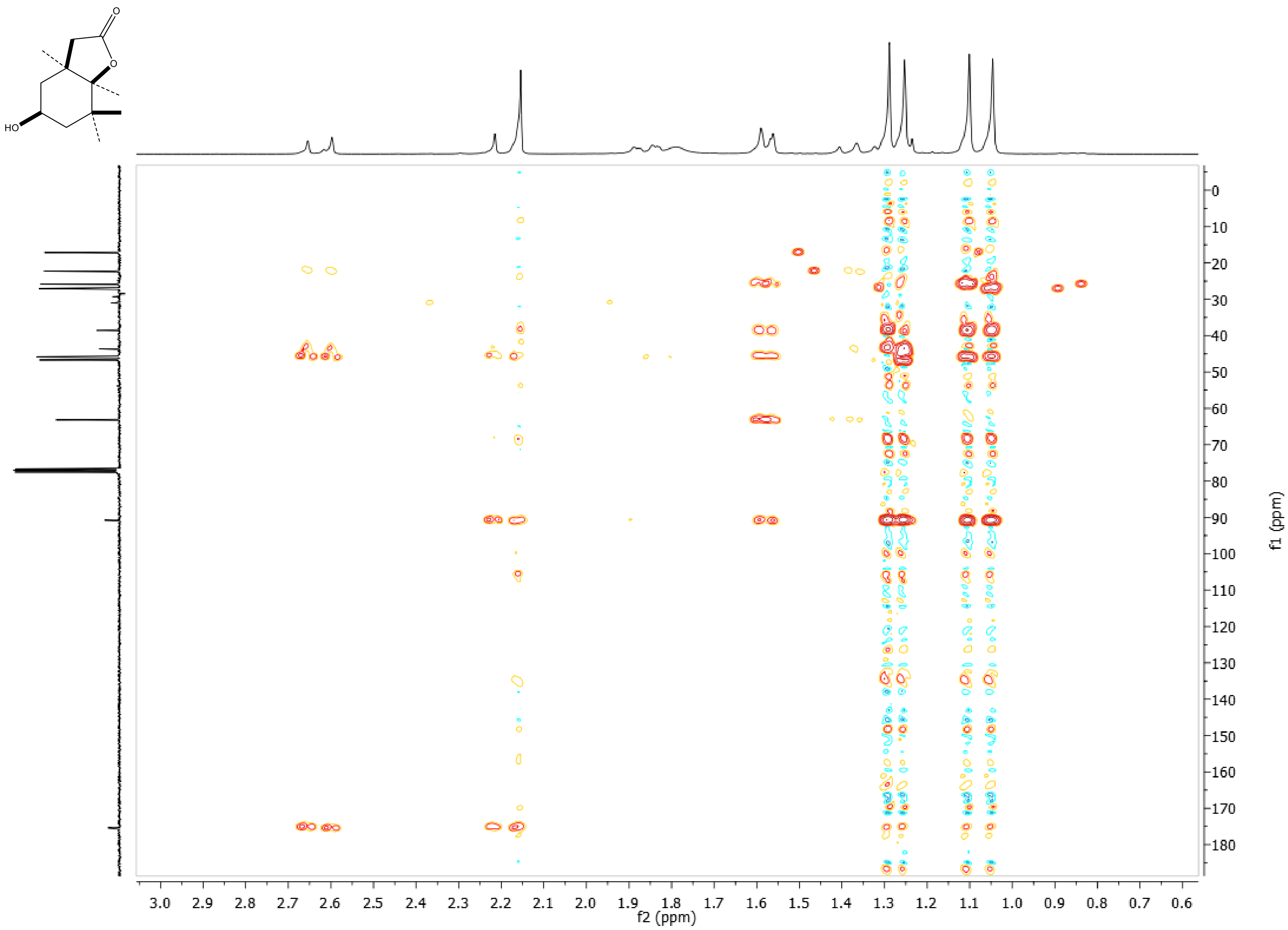

Figure S8. HMBC lactone 3

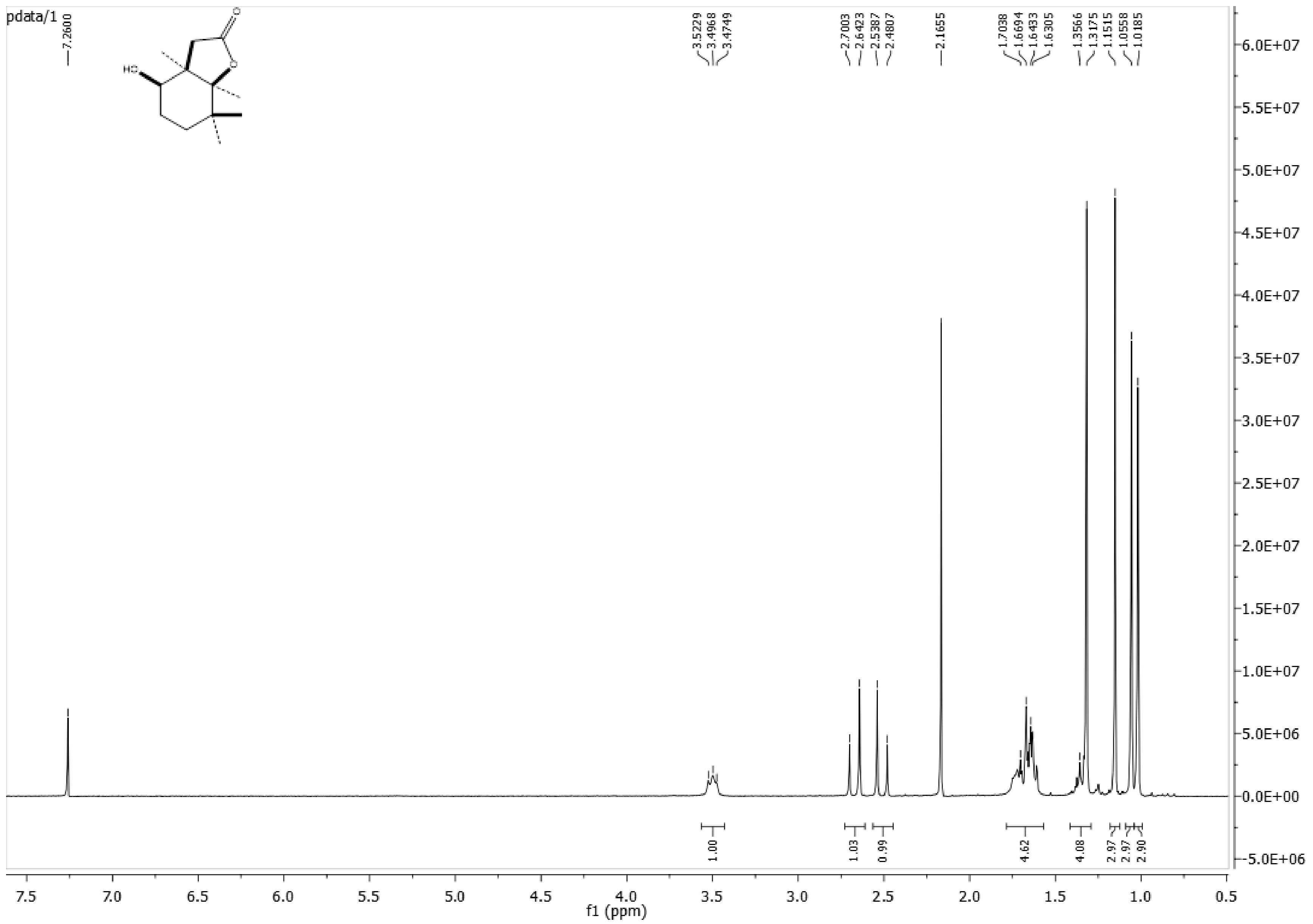

Figure S9. ^{1}H NMR lactone 4

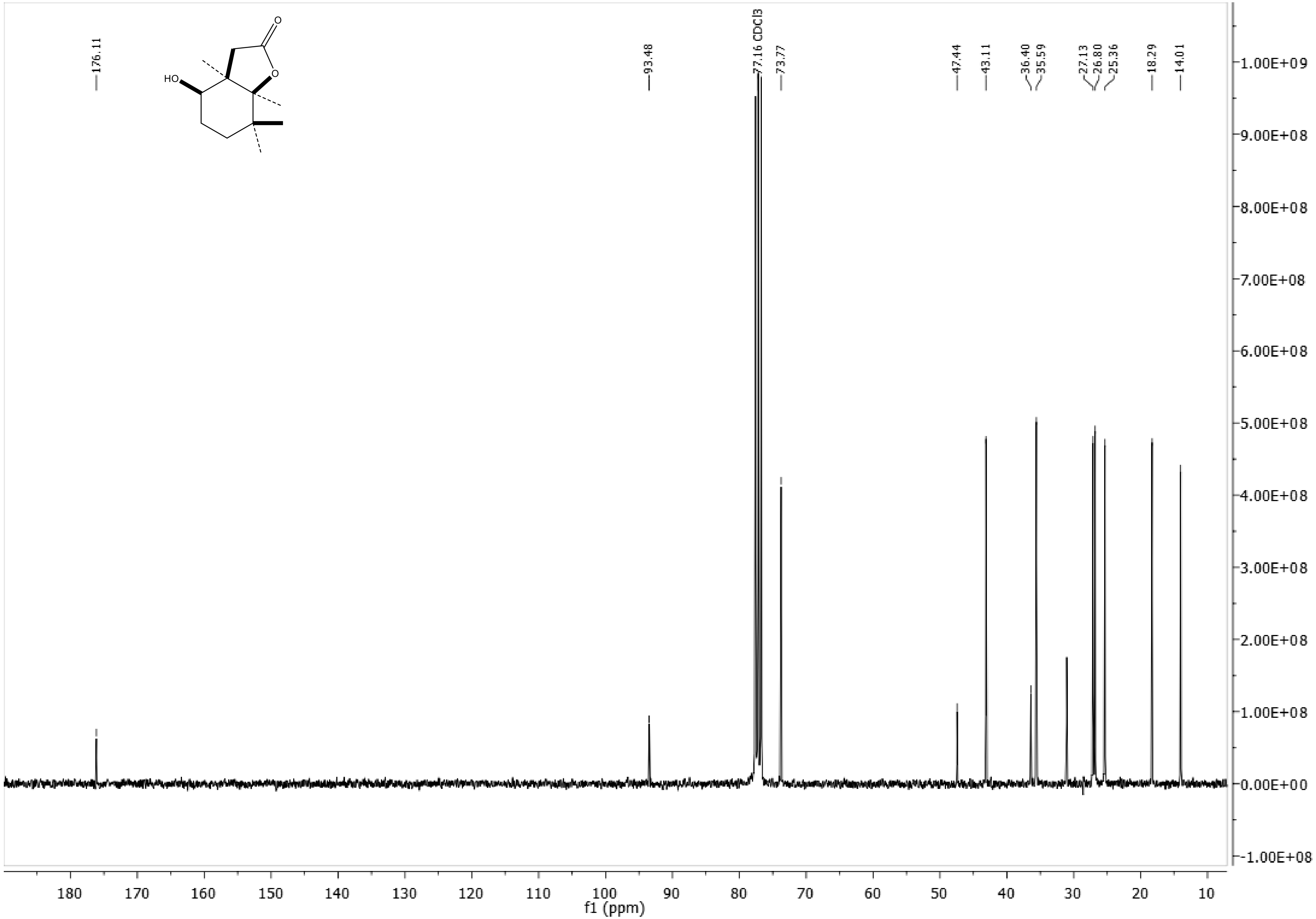

Figure S10. ^{13}C NMR lactone 4

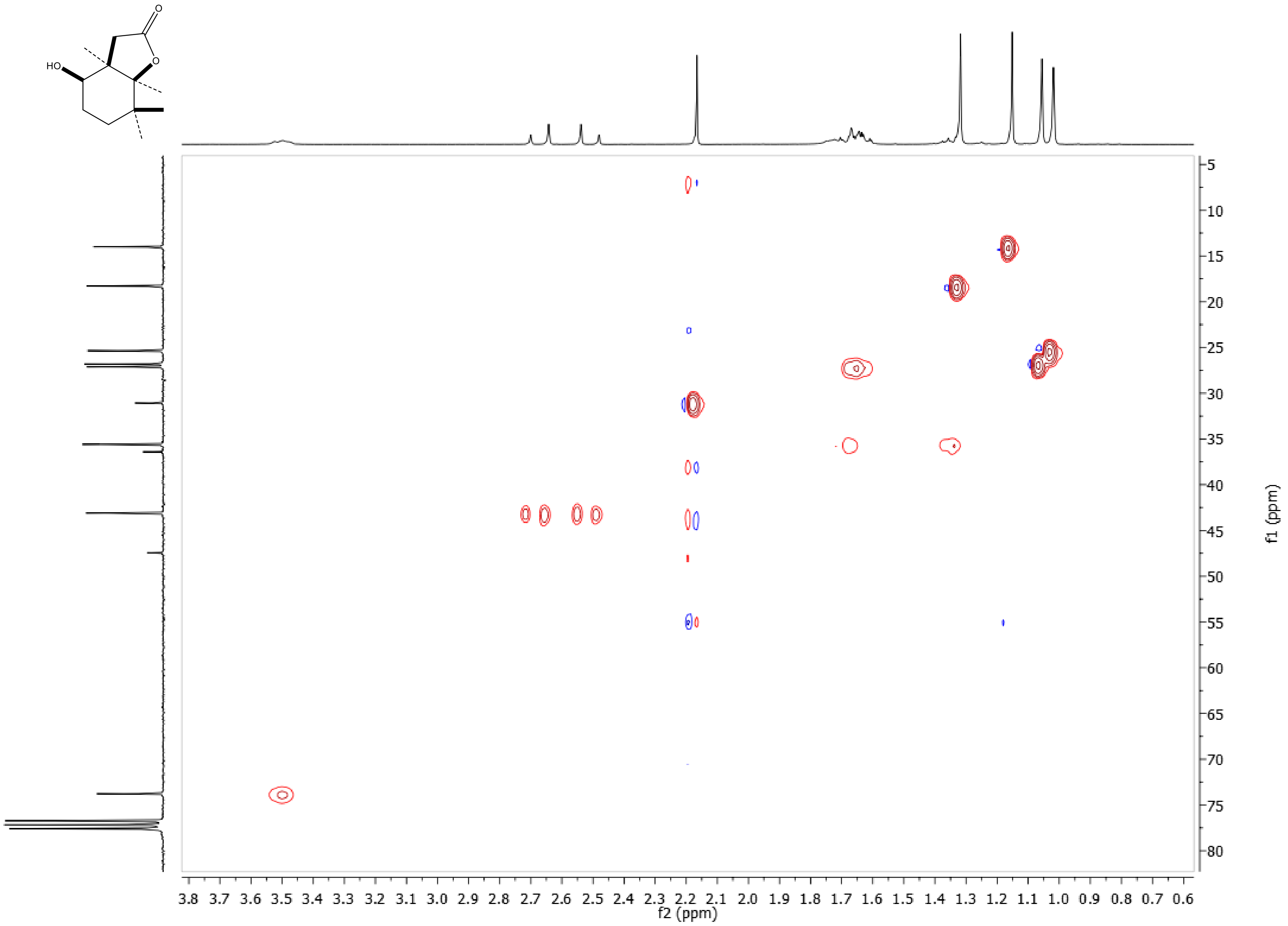

Figure S11. HMQC lactone 4

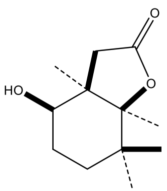

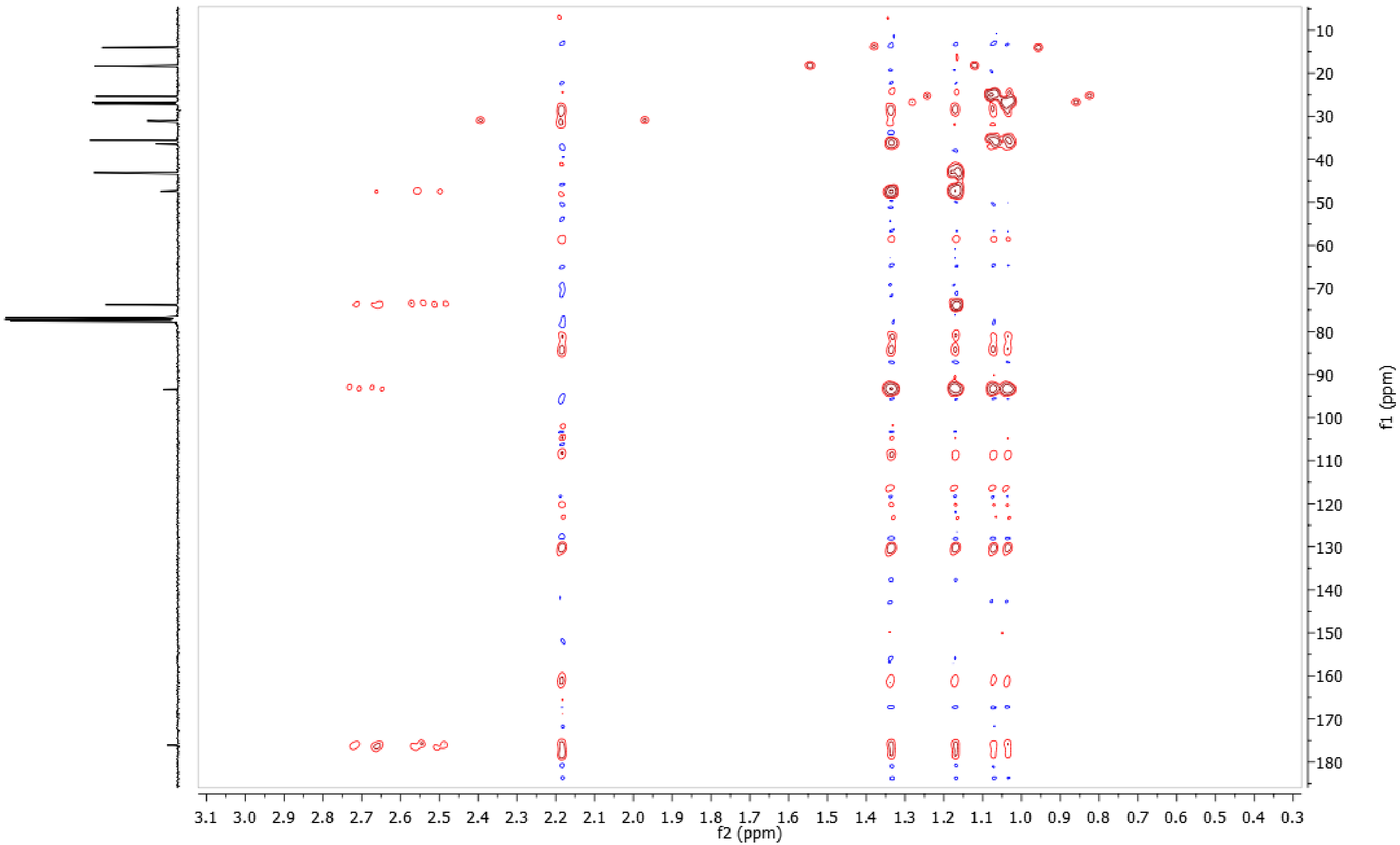

Figure S12. HMBC NMR lactone 4
